# Effect of *CYP3A5* on the Once-Daily Tacrolimus Conversion in Stable Liver Transplant Patients

**DOI:** 10.3390/jcm9092897

**Published:** 2020-09-08

**Authors:** Jong Man Kim, Je Ho Ryu, Kwang-Woong Lee, Suk Kyun Hong, Kwangho Yang, Gyu-Seong Choi, Young-Ae Kim, Ju-Yeun Lee, Nam-Joon Yi, Choon Hyuck David Kwon, Chong Woo Chu, Kyung-Suk Suh, Jae-Won Joh

**Affiliations:** 1 Department of Surgery, Samsung Medical Center, Sungkyunkwan University School of Medicine, Seoul 13557, Korea; yjongman21@gmail.com (J.M.K.); gyuseong.choi@samsung.com (G.-S.C.); jw.joh@samsung.com (J.-W.J.); 2 Department of Surgery, Pusan National University Yangsan Hospital, Pusan National University School of Medicine, Busan 46241, Korea; ryujhhim@hanmail.net (J.H.R.); ykrep@hanmail.net (K.Y.); 3 Department of Surgery, Seoul National University College of Medicine, Seoul 08826, Korea; nobel1210@naver.com (S.K.H.); gsleenj@hanmail.net (N.-J.Y.); kssuh2000@gmail.com (K.-S.S.); 4 Department of Pharmacy, Seoul National University Hospital, Seoul 03080, Korea; kimya@snuh.org; 5 College of Pharmacy and Research Institute of Pharmaceutical Sciences, Seoul National University, Seoul 04213, Korea; jypharm@snu.ac.kr; 6 Department of Surgery, Digestive Disease and Surgery Institute, Cleveland Clinic, Cleveland, OH 9500, USA; chd.kwon@gmail.com; 7 Department of Hepatobiliary Surgery and Liver Transplantation, Good Gang-An Hospital, Busan 613-815, Korea; liversurgeon@hanmail.net

**Keywords:** pharmacokinetics, immunosuppression, tacrolimus

## Abstract

Cytochrome P450 (*CYP*) *3A5* polymorphism influences tacrolimus metabolism, but its effect on the drug pharmacokinetics in liver transplant recipients switched to once-daily extended-release formulation remains unknown. The aim of this study is to analyze the effect of *CYP3A5* polymorphism on liver function after once-daily tacrolimus conversion in liver transplant patients. A prospective open-label study included 60 stable liver transplant recipients who underwent 1:1 conversion from twice-daily tacrolimus to once-daily tacrolimus. All participants were genotyped for *CYP3A5* polymorphism. The study was registered at ClinicalTrials.gov (NCT 02882113). Twenty-eight patients were enrolled in the *CYP3A5* expressor group and 32 in the non-expressor group. Although there was no statistical difference, incidence of liver dysfunction was higher in the expressor group than in the non-expressor group when converted to once-daily extended-release tacrolimus (*p* = 0.088). No biopsy-proven acute rejection, graft failure, and mortality were observed in either group. The decrease in dose-adjusted trough level (−42.9% vs. −26.1%) and dose/kg-adjusted trough level of tacrolimus (−40.0% vs. −23.7%) was significantly greater in the expressor group than in the non-expressors after the conversion. A pharmacokinetic analysis was performed in 10 patients and tacrolimus absorption in the non-expressor group was slower than in the expressor group. In line with this observation, the area under the curve for once-daily tacrolimus correlated with trough level (Cmin) in the non-expressors and peak concentration (Cmax) in the expressors. *CYP3A5* genotyping in liver transplant recipients leads to prediction of pharmacokinetics after switching from a twice-daily regimen to a once-daily dosage form, which makes it possible to establish an appropriate dose of tacrolimus.

## 1. Introduction

Tacrolimus is an effective immunosuppressant, and its use in liver transplantation (LT) is well established [1]. However, the drug has a narrow therapeutic window, and its pharmacokinetics and pharmacodynamics vary considerably at both intraindividual and interindividual levels; hence, establishing an empirical dosage regimen may pose a challenge [2,3,4]. Therapeutic drug monitoring is clinically important as it may contribute to better efficacy of the treatment, lower rejection rates, and fewer adverse reactions [3]. Underdosing of tacrolimus may lead to rejection or graft failure, whereas overdosing increases the risk of nephrotoxicity, neurotoxicity, infection, hypertension, post-transplant diabetes, and malignancy [2,3]. Given a poor correlation between the dosage of tacrolimus and its blood concentration, the dose is often adjusted based on the latter parameter to achieve an optimal balance between the efficacy and toxicity of the drug.

As a result of recent advances in pharmacogenomics, several single nucleotide polymorphisms in the intron 3 of *CYP3A5* were identified and were shown to correlate with the expression of the gene and enzymatic activity of the product it encodes [4]. The intra- and interindividual variability in tacrolimus pharmacokinetics and pharmacodynamics is partly associated with the polymorphism of the genes for cytochrome P450 enzymes, *CYP3A4* and *CYP3A5*, and the efflux transporter P-glycoprotein (P-gp); all these molecules have been implicated in altered absorption and metabolism of tacrolimus [4]. Polymorphism at a cryptic splice site may result in either the presence (expressor, *1/*1 and *1/*3) or absence (non-expressor, *3/*3) of *CYP3A5* [4]. The frequencies of *CYP3A5* polymorphism differ depending on race [4,5], with the CYP3A5 expressors (i.e., *1/*1 or *1/*3) and *MDR1 C3435T* wild-type C allele carriers (i.e., *CC* or *CT*) being more prevalent among Asians (51% and 62.1%, respectively) than in Caucasians (10% and 43.4%, respectively) [6].

Several studies demonstrated that the expression of *CYP3A5* results in a lower tacrolimus exposure, and hence *CYP3A5* expressors might require a higher dose of the drug than non-expressors [2,3,7,8]. While we still know little about the effect of the *CYP3A5* genotype on patient performance post-transplant, it has been shown to affect tacrolimus pharmacokinetics. Furthermore, several studies showed that polymorphism of various genes might be associated with the occurrence of adverse effects and graft survival in renal transplant patients [2,8,9]. Given those findings, the effect of the *CYP3A5* genotype on tacrolimus pharmacokinetics should also be considered in liver transplant recipients.

A once-daily extended-release formulation (Advagraf^®^) has been approved in many countries since 2007. Several studies have demonstrated that in stable patients after LT, conversion from twice-daily to once-daily tacrolimus was well-tolerated, safe, and convenient [10,11]. However, despite the similar pharmacokinetics of the twice-daily and once-daily tacrolimus, some patients experienced adverse events, including liver dysfunction, after the conversion [12].

To the best of our knowledge, none of the previous studies analyzed a link between *CYP3A5* polymorphism and the changes in tacrolimus pharmacokinetics after conversion from the twice-daily regimen to a once-daily formulation. Therefore, the aim of this study was to compare the incidence of liver dysfunction in stable liver transplant recipients, both *CYP3A5* expressors and non-expressors, after conversion to once-daily expanded-release tacrolimus (Advagraf^®^), and to analyze the effect of the *CYP3A5* genotype on the pharmacokinetics of both a twice-daily regimen and a once-daily tacrolimus formulation.

## 2. Materials and Methods

### 2.1. Study Design

This prospective, multicenter, open-label, exposure-variable study was carried out at the Samsung Medical Center (Seoul, Korea), Seoul National University Hospital (Seoul, Korea), and Yangsan Pusan National University Hospital (Yangsan, Busan) between August 2016 and June 2018. The study was carried out in accordance with the Declaration of Helsinki and was registered at ClinicalTrials.gov (NCT 02882113). The protocol of the study was approved by the Institutional Review Boards at the Samsung Medical Center (IRB No. 2015-11-140), Seoul National University Hospital (IRB No. H-1510-097-712), and Yangsan Pusan National University Hospital (IRB No. 04-2015-038). All participants provided written informed consent to participate in the study.

### 2.2. Patients

The inclusion criteria of the present study were: age ≥19 years, more than 6 months after liver transplantation and within 3 years, the use of twice-daily tacrolimus at screening, stable renal function (serum creatinine level < 2.0 mg/dL), serum aspartate aminotransferase (AST) and alanine aminotransferase (ALT) ≤32 IU/L [13], and maintenance of the same immunosuppressive dosing regimen for ≥2 weeks before the enrollment. The patients who received any drugs known to interfere with tacrolimus pharmacokinetics and those enrolled in other immunosuppressant study protocols were not eligible for the study. Other exclusion criteria were: trough level of tacrolimus at screening <2 ng/mL, an acute rejection episode within 90 days before the enrollment, other organ transplant, renal dysfunction (serum Cr ≥ 2 mg/dL or estimated glomerular filtration rate, eGFR < 30mL/min), patients received cyclosporin or mammalian target of rapamycin (mTOR) inhibitors, clinically significant infection, a history of malignancy other than hepatocellular carcinoma or skin cancer, recurrent hepatitis B virus or hepatitis C virus infection, liver dysfunction, pregnancy, or unstable concurrent medical condition.

This was not a hypothesis-driven study, but a pilot study to investigate the effect of *CYP3A5* polymorphism on liver function and trough level of tacrolimus after conversion to the once-daily extended-release tacrolimus formulation. The study patients were grouped based on the expressions of *CYP3A5* polymorphism (expressors vs. non-expressors). The minimum required sample size was 30 patients per group. This number was based on a general assumption that the variance for a sample of at least 30 reflects the population variance quite accurately, and hence statistical testing that does not target a specific hypothesis is valid [14]. Therefore, our goal was to recruit a total of 60 patients, 30 per group. The dropout rate was not considered in the safety-oriented analysis. In addition, we performed pharmacokinetic analysis for a total of 10 patients with 5 patients per group in the order of patients who agreed to pharmacokinetic analysis.

### 2.3. Tacrolimus Concentrations

Once-daily tacrolimus was administered at 8 A.M., and the dose was adjusted according to the daily trough level of the drug (C0 or Cmin). The trough levels of tacrolimus were measured by liquid chromatography-tandem mass spectrometry using a Waters 2795 Alliance HT system (Waters Ltd., Watford, UK) and a Quattro micro API tandem mass spectrometer (Micromass, Manchester, UK). Before and after the conversion, all tacrolimus doses were adjusted according to the trough level of the drug, to obtain a therapeutic window of 2–8 ng/mL.

### 2.4. Genotyping of Cytochrome P450 3A5

Genomic DNA was extracted from whole blood samples using a QIAamp DNA Mini kit (Qiagen Inc., Germantown, MD, USA), and stored at −20 °C. The genotypes of the *CYP3A5* gene (*6986 A>G* in intron 3) and the *ABCB1* gene (*1236C>T* in exon 12 and *3435C>T* in exon 26) were determined using the TaqMan allelic discrimination assay. Primers and probes were designed with Primer Express Software Version 3.0 (Applied Biosystems, Foster City, CA, USA). The polymerase chain reaction (PCR) mixture included 50 ng of genomic DNA in a 1 μL volume, along with the following reagents: FAM and TET probes (5 pmol/μL, respectively), primers (20 pmol/μL for sense and antisense primers, respectively), and 2X TaqMan Universal PCR Master Mix (Applied Biosystems). PCR cycling reactions were conducted in an ABI 9700 PCR system (Applied Biosystems); the reactions consisted of initial 10-min denaturation at 95 °C, followed by 40 cycles of 15-s denaturation at 95 °C and 1-min annealing and extension at 60 °C. The results were analyzed with the ABI 7900HT Sequence Detection System (Applied Biosystems). To control the genotyping quality, 10% of the samples were randomly selected for retyping with a double-blind check. Genetic polymorphisms were analyzed on the day of transplantation. The study patients were blinded to the genetic polymorphisms they carried.

Considering the low frequency of the CYP3A5*1 allele, its carriers (CYP3A5*1/*1 or CYP3A5*1/*3) were selected first and classified as the “expressors”. The patients who carried the CYP3A5*3/*3 genotype, responsible for the lack of *CYP3A5* expression, were selected second, and were classified as the “non-expressors”.

### 2.5. Immunosuppression

Immunosuppressive therapy after LT was based on the combination of calcineurin inhibitor (tacrolimus) and mycophenolate mofetil (MMF). Two types of twice-daily tacrolimus were used before the conversion: the reference tacrolimus product (Prograf^®^; Astellas Pharma, Tokyo, Japan) and the generic formulation of tacrolimus (Tacrobell^®^; Chong Kun Dang Pharma, Seoul, Korea). All patients were converted to once-daily tacrolimus (Advagraf^®^, Astellas Pharma, Inc., Deerfield, IL, USA) on a 1:1 mg basis for the total daily dose. However, some dose adjustments were permitted depending on the patient’s condition, and other immunosuppressants were allowed to be used according to standard practice. The serum trough levels of tacrolimus and clinical assessments for safety and rejection were completed four weeks after the conversion; then, parameters were evaluated routinely according to the patient’s follow-up schedule. The doses of tacrolimus at baseline and during follow-up were adjusted on an individual basis according to the serum trough level of the drug.

### 2.6. Endpoints

The primary endpoint of this study was the incidence of liver dysfunction that required discontinuation or dosage modification of tacrolimus from registration to six months. The liver dysfunction was defined as an AST or ALT level greater than two times the upper limit of normal. Liver function tests elevation by biliary or vascular complications is not considered as liver dysfunction. Liver biopsy was not routinely performed in all patients with liver function abnormalities. The secondary endpoints were tacrolimus trough level changes and the incidence of acute rejection. The rejection was defined according to the Banff criteria for liver biopsy [15,16]. Additionally, the changes in pharmacokinetic parameters of tacrolimus before and after the conversion to the once-daily extended-release formulation were analyzed.

### 2.7. Pharmacokinetic Analysis

Blood sampling for pharmacokinetics of twice-daily tacrolimus was performed within 3 weeks. After taking once-daily tacrolimus, blood sampling was performed between 1 and 2 weeks to analyze pharmacokinetics. Thirteen blood samples (3 mL each) were collected at 0 (pre-dose) and 0.5, 1, 1.5, 2, 3, 4, 6, 9, 12, 16, 20, and 24 h after the morning dose. All blood samples were collected into ethylenediaminetetraacetic acid-containing Vacutainer^®^ tubes and were stored at 4 °C until the analysis was carried out on the same day. The trough levels (C0 and Cmin) were measured just before tacrolimus administration; peak tacrolimus concentration (Cmax) for each subject was obtained directly from the raw data. The area under the curve (AUC) was estimated using the linear trapezoidal method from hours 0 to 24.

### 2.8. Statistical Analysis

Statistical analyses were conducted using SPSS software (version 22.0, SPSS Inc., Chicago, IL, USA). The data are expressed as medians and ranges or as frequencies (percentages). All tests were two-tailed, and *p* < 0.05 was considered significant. The values of continuous variables in the expressors and non-expressors were compared using the Mann–Whitney *U* test, and the Wilcoxon test was used to compare the value of continuous variables within the same group. The distributions of categorical variables were compared with the Fisher exact test. The effect of *CYP3A5* polymorphism on changes in tacrolimus exposure after the conversion to the once-daily formulation was tested during the analysis of covariance (ANCOVA).

## 3. Results

### 3.1. Patient Characteristics

**Figure 1:**
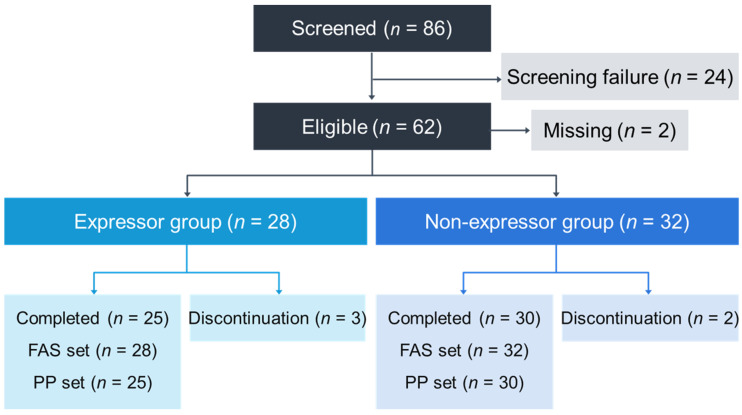
Study population. FAS, full analysis set; PP, per-protocol set.

Eighty-six patients were screened for enrollment to this study. A total of 24 patients were excluded at the screening because of a surplus of *CYP3A5* non-expressors (as stated above, the target size of this group was defined as approximately 30). As another two patients were excluded due to an overdose of tacrolimus after the conversion to the once-daily extended-release formulation, the study included 60 patients, with 28 expressors and 32 non-expressors among them (Figure 1). The full analysis set (FAS) included all eligible patients who received ≥1 dose of once-daily extended-release tacrolimus, with the patients analyzed according to the treatment allocation. The per-protocol (PP) set included all patients who completed the study without a major protocol deviation.

The non-expressor group included a significantly higher proportion of men than the expressor group (77.4% vs. 42.9%, *p* = 0.008). The two study groups did not differ significantly in terms of other clinical characteristics or in the time elapsed from the liver transplantation to the study recruitment (Table 1).

### 3.2. Efficacy

**Figure 2:**
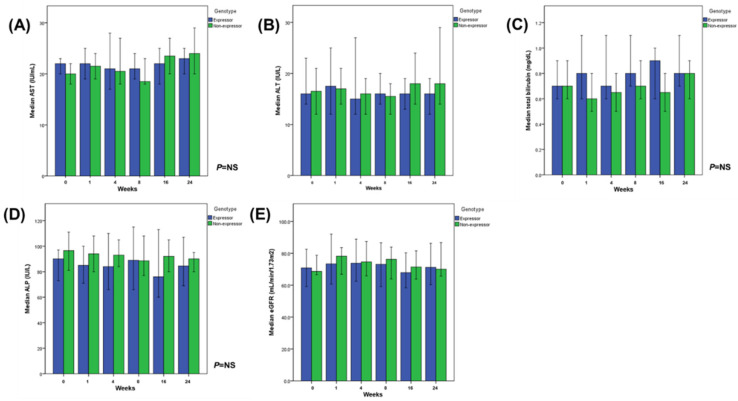
Laboratory parameters of *CYP3A5* expressors and non-expressors determined during regular control visits in per-protocol set (medians with 95% confidence interval). (**A**) Aspartate aminotransferase (AST), (**B**) Alanine aminotransferase (ALT), (**C**) total bilirubin, (**D**) Alkaline phosphatase (ALP), and (E) eGFR.

In the full analysis set (FAS), the incidence of liver dysfunction after the conversion to once-daily extended-release tacrolimus was 17.9% in the expressor group (*n* = 5) versus 3.1% (*n* = 1) in the non-expressor group (*p* = 0.088). In the per-protocol (PP) set, the incidence of liver dysfunction in the expressor group was higher than in the non-expressor group (12.0% vs. 0%, *p* = 0.088). No biopsy-proven acute rejection, graft failure, and mortality were observed in either group during the study period (Table 1). The expressor and non-expressor groups did not differ significantly in terms of serum estimated glomerular filtration rate (eGFR) levels and liver parameters, such as AST, ALT, total bilirubin, and alkaline phosphatase (ALP), during the follow-up (Figure 2).

### 3.3. Tacrolimus Dose Adjustments after the Conversion

Eight patients from the expressor group (28.6%) and seven patients from the non-expressor group (21.9%) required an increase in the dose of extended-release tacrolimus during the study period. The dose of tacrolimus was decreased in two patients from the expressor group (7.1%) and in three patients from the non-expressor group (9.4%). The frequency of tacrolimus dose adjustment did not differ significantly between the study groups (*p* = 0.787).

### 3.4. Tacrolimus Exposure before and after the Conversion

**Figure 3:**
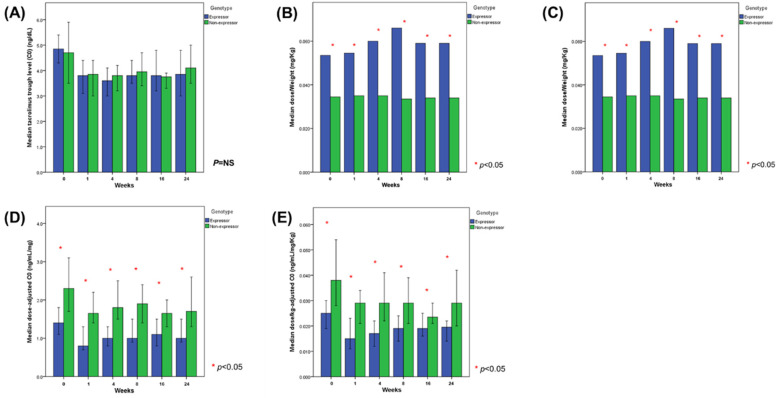
Tacrolimus exposure in *CYP3A5* expressors and non-expressors determined during regular control visits in per-protocol set (medians with 95% confidence interval). (**A**) trough level of tacrolimus, (**B**) daily tacrolimus dose, (**C**) tacrolimus dose/weight, (**D**) dose-adjusted C0, and (**E**) dose/kg-adjusted C0.

The main parameters of tacrolimus exposure determined in both study groups (expressors and non-expressors) after administration of the twice-daily and once-daily formulations are summarized in Table 2, and the blood concentration–time profiles of tacrolimus are shown in Figure 3. After the administration of tacrolimus, the median daily dose of the drug and the median dose/weight were significantly higher in the expressor group (*p* < 0.001). Despite this, the study groups did not differ significantly in terms of the median tacrolimus trough levels during the follow-up. The median dose-adjusted trough level (C0/dose) and dose/kg-adjusted trough level (C0/dose/kg) were significantly lower in the expressor group than in the non-expressor group (*p* < 0.001).

After the conversion to once-daily extended-release tacrolimus, median trough level of the drug (C0) decreased by nearly 25%; the change was similar regardless of the *CYP3A5 genotype* (−24.0% in the expressor group vs. −25.5% in the non-expressor group). Moreover, a decrease in the median dose-adjusted trough level and dose/kg-adjusted trough level was observed, by 42.9% and 40.0% in the expressor group, respectively, and by 26.1% and 23.7% in the non-expressor group, respectively. The decrease in the dose-adjusted trough level and dose/kg-adjusted trough level was significantly greater in the expressor group than in the non-expressor group (*p* < 0.001 and *p* < 0.001, respectively).

### 3.5. Pharmacokinetics before and after the Conversion

**Figure 4:**
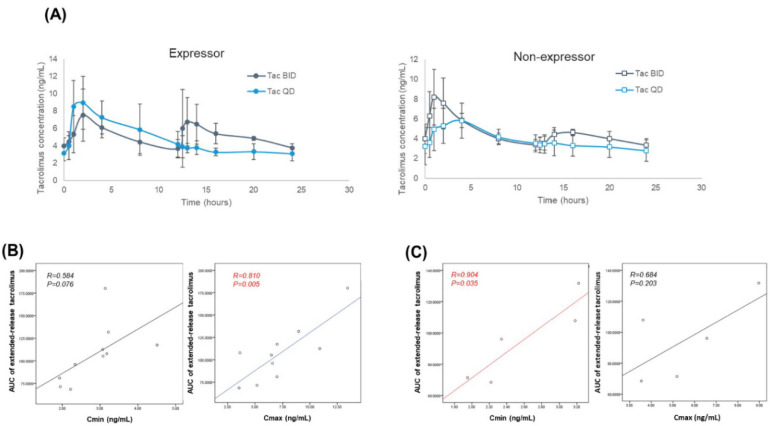
(**A**) 24-h arithmetic mean whole blood tacrolimus concentrations (ng/mL) and standard deviation for the twice-daily and once-daily formulations; (**B**) Correlation between the area under the curve (AUC) and Cmin or Cmax in the expressor group after conversion to once-daily extended-release tacrolimus; (**C**) Correlation between the area under the curve (AUC) and Cmin or Cmax in the non-expressor group after conversion to once-daily extended-release tacrolimus.

Pharmacokinetic findings of this study are summarized in Table 3. Mean whole blood concentrations of tacrolimus before and after the conversion are depicted in Figure 3. No statistically significant differences were found in the area under the curve (AUC), Cmin, Cmax, dose-adjusted Cmin, dose-adjusted Cmax, dose-adjusted AUC, dose/kg-adjusted Cmin, dose/kg-adjusted Cmax, and dose/kg-adjusted AUC between the twice-daily tacrolimus and once-daily tacrolimus groups. The median dose of tacrolimus was higher in the expressor group than in the non-expressor group, while median dose/weight, Cmax in twice-daily tacrolimus/dose, AUC in twice-daily tacrolimus, and dose/kg-adjusted Cmax in twice-daily tacrolimus in the expressors were significantly lower than in the non-expressors (Table 3). Median AUC values for the twice-daily tacrolimus and once-daily tacrolimus were higher in the expressor group than in the non-expressor group, but the differences were not significant. In the expressor group, the Cmax for once-daily tacrolimus was higher than the Cmax for twice-daily tacrolimus, whereas an inverse relationship was observed in the non-expressor group. In line with these findings, significant correlations were found between the Cmin and AUC for once-daily tacrolimus in the non-expressor group, and between the Cmax and AUC for once-daily tacrolimus in the expressor group (Figure 4).

### 3.6. Safety

**Figure 5:**
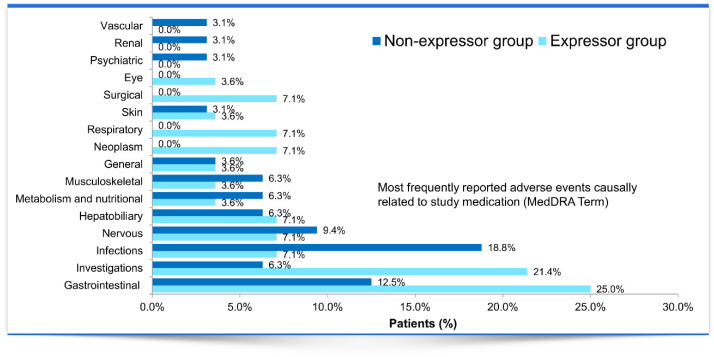
Adverse event profiles in the expressors and non-expressors (Full Analysis Set) after conversion from twice-daily to once-daily extended-release tacrolimus.

Adverse events (AEs) are summarized in Table 4. A total of 71 AEs occurred in 35 out of 60 (58.3%) patients included in the FAS. The distributions of AEs in the expressor and non-expressor groups did not differ significantly (*p* = 0.796). Most AEs, 82.9% in the expressor group and 63.3% in the non-expressor group, were mild. However, the incidence of moderate-severity AEs in the expressor group was lower than in the non-expressor group (14.6% vs. 33.3%). In the expressor group, 24.4% of the AEs had a probable or possible relationship to the study drug, and three patients experienced AEs that led to drug discontinuation. No AEs related to the study drug or requiring its discontinuation were recorded in the non-expressor group. While the incidence of AEs in the investigations and gastrointestinal system was higher in the expressor group than in the non-expressor group, the study groups did not differ significantly in the incidence of AEs involving the other organ systems (Figure 5). Serious AEs involving the nervous system (*n* = 1), investigations (*n* = 2), and gastrointestinal system (*n* = 3) occurred solely in the expressor group. Additionally, severe infectious complications were not developed in the patients.

## 4. Discussion

The safety and efficacy of twice-daily and once-daily tacrolimus in liver transplant recipients were shown to be similar [3,11,17,18,19]. Several previous studies demonstrated that liver dysfunction is the most common adverse event after converting from twice-daily to once-daily tacrolimus [12]. In our present study, the incidence of liver dysfunction in stable liver transplant recipients converted to once-daily extended-release tacrolimus was higher in the *CYP3A5* expressor group than in the non-expressor group. However, the between-group difference was not statistically significant. None of the study patients experienced acute rejection or graft failure, and no mortality was observed in our series. In previous studies, *CYP3A5* expression was shown to be associated with a significant increase in the risk of biopsy-proven acute rejection and tacrolimus-induced nephrotoxicity at three months post-transplant [20], and with a decrease in eGFR at three months after renal transplantation [21]. However, our present study did not demonstrate a significant difference in renal function in *CYP3A5* expressors and non-expressors.

After 1:1 conversion from twice-daily to once-daily extended-release tacrolimus, the trough level of the drug in stable adult liver transplant recipients decreased by nearly 25%. Up to 25% of the patients required a tacrolimus dose increase after the conversion. These findings are consistent with the results of other once-daily tacrolimus conversion studies in LT patients [10,11,12,19]. A 15–20% increase in the daily dose of tacrolimus has been suggested in order to achieve the same target trough level of the drug following the conversion [22].

*CYP3A5* expression was shown to influence tacrolimus exposure, with the exposure in *CYP3A5* expressors receiving either twice-daily tacrolimus or once-daily tacrolimus being lower than in the non-expressors [6,7,8,17]. *CYP3A5* expressors carry the *1 variant which encodes the functional enzyme responsible for the metabolism of tacrolimus; therefore, they may require a higher dose of tacrolimus than the non-expressors to achieve the target trough level of the drug [3,4,5]. Our present study demonstrated that *CYP3A5* polymorphism influenced both tacrolimus dose and trough level of the drug; the median dose of tacrolimus both before and after switching to the once-daily extended-release formulation was significantly higher in the expressor group than in the non-expressors. This observation is also consistent with the results of previous studies in which *CYP3A5* expressors, both adult and pediatric patients, required higher doses of tacrolimus due to the higher oral clearance of the drug [9,23]. Our present study showed that the decrease in the trough level of tacrolimus was greater in the expressor group than in the non-expressors, also after adjustment for patient weight and tacrolimus dose. The *CYP3A5* genotype is known to play a role in determining the effect of interacting drugs, such as fluconazole, on tacrolimus pharmacokinetics [5]. However, it needs to be stressed that our study did not analyze the issue of drug interaction, and all patients who received agents that might potentially interact with tacrolimus were excluded from the analysis.

While we did not find a significant difference in the incidence of AEs in the expressors and non-expressors, the frequency of drug-related AEs was higher in the former group. This was likely associated with a higher dose of tacrolimus received by the expressors. In view of this observation, a higher dose of tacrolimus might be a confounder in previous studies analyzing the link between the *CYP3A5* genotype and nephrotoxicity risk, especially given that our present study did not demonstrate a significant *CYP3A5* genotype-related difference in the occurrence of renal dysfunction. In a recent study, Korean adult liver transplant recipients with *CYP3A5* expression presented with low peripheral blood CD4+ adenosine triphosphate (ATP) immune response activity despite maintaining a constant concentration of tacrolimus, and suffered from infectious complications [24]. In contrast, the incidence of infectious complications in our present study was lower in the expressor group than in the non-expressor group (7.1% vs. 18.3%), and no severe infections were recorded among the expressors.

The correlation between the Cmin of tacrolimus and the effects of the drug is known to be stronger than the dose–effect correlation [25]. Due to a strong correlation between Cmin and systemic exposure (AUC), the dose of tacrolimus can be tailored using the Cmin level as a surrogate marker of exposure [3]. Monitoring of Cmin is mandatory to minimize the risk of rejection (Cmin below the target range), as well as to reduce the risk of nephrotoxicity, and, to a lesser extent, neurotoxicity (Cmin above the target range) [3,17]. In the present study, the AUC for twice-daily tacrolimus did not differ significantly from the AUC for once-daily tacrolimus, despite a lower Cmin for the latter. This implies that the same target trough level of tacrolimus cannot be used to predict the efficacy of the drug (AUC) after the conversion, especially among the expressors. In the non-expressor group, Cmin correlated significantly with AUC, whereas a significant correlation between Cmax and AUC was observed in the expressor group. Genetic polymorphisms are known to influence drug metabolism and have been implicated as a cause of individual variability in drug pharmacokinetics. A difference in the *CYP3A5* protein expression level in the small intestine, and replacement of croscarmellose with ethylcellulose may influence the oral bioavailability of tacrolimus in both initial exposure and steady state. Therefore, the diffusion rate of tacrolimus after administration of its once-daily formulation leads to a prolonged release of the drug. In our present study, the absorption of once-daily tacrolimus in *CYP3A5* non-expressors was slower compared with the twice-daily formulation.

In a previous study, conversion from twice-daily to once-daily tacrolimus was associated with a 21% decrease in the median drug exposure in *CYP3A5* expressors. Based on that observation, approximately a 1.25-fold increase in total daily tacrolimus dose was recommended in *CYP3A5* expressors after switching to the once-daily formulation, to maintain the same level of tacrolimus exposure [26]. However, the results of our present study imply that the dose adjustment might not be necessary since, in the expressor group, the AUC after conversion to the once-daily extended-release tacrolimus did not differ significantly from that after the conversion, and unlike in the non-expressors, the AUC correlated with Cmax, rather than with Cmin.

This study has several limitations. First, the sample size was relatively small, and the follow-up period was quite short. Hence, further large-scale studies are needed to determine whether the events occurring during the post-transplant period might have an adverse effect on the long-term outcome in liver transplant recipients. Second, although we compared the incidence of liver dysfunction in the expressor and non-expressor groups, reducing the drug concentration in this study increased the tacrolimus dose given by the physician to maintain an adequate trough level, which limits the analysis. Therefore, it was difficult to determine the true incidence of liver dysfunction. Third, our study was conducted with Koreans. Therefore, our results cannot be generalized to patients in Western countries. Fourth, the pharmacokinetic analysis included only ten patients, and previous studies documented a considerable intra- and interpatient variability in the pharmacokinetics of tacrolimus delivered in either twice-daily or once-daily formulations [22,27,28]. Hence, the effects of *CYP3A5* gene polymorphism on tacrolimus pharmacokinetics need to be verified in a larger group of liver transplant recipients. Fifth, we did not know the *CYP3A5* gene status of liver donors. Unlike in other organ transplantations, the liver transplant does not need to share the genetic background of the recipient. As the activity of most drug-metabolizing enzymes is very elevated in the liver, polymorphism of the *CYP3A5* gene, whether in the recipient or the donor, would likely contribute to individual variance in drug pharmacokinetics.

## 5. Conclusions

The results of the present study suggest that *CYP3A5* genotyping in liver transplant recipients leads to prediction of pharmacokinetics after switching from a twice-daily regimen to a once-daily dosage form, which makes it possible to establish an appropriate dose of tacrolimus. After the conversion, *CYP3A5* expressors showed a more evident decrease in the trough level of tacrolimus (Cmin) than the non-expressors. However, the pharmacokinetic analysis did not show a significant difference in the AUC before and after the conversion. The AUC in *CYP3A5* expressors switched to once-daily tacrolimus correlated with Cmax, rather than with Cmin. This implies that *CYP3A5* expression might have a greater influence on the pharmacokinetics of once-daily tacrolimus than the twice-daily tacrolimus formulation.

The clinical relevance of the findings presented above needs to be verified in further large-scale studies analyzing various pharmacogenetic strategies for tacrolimus dosing and the effect of *CYP3A5* genetic polymorphism on long-term outcomes in liver transplant recipients.

## 6. Patents

This section is not mandatory, but may be added if there are patents resulting from the work reported in this manuscript.

## Figures and Tables

**Table 1:** Characteristics and efficacy of the study patients stratified according to *CYP3A5* expression.

	Expressor Group (*n* = 28)	Non-Expressor Group (*n* = 32)	*p*-Value
**Donor**			
Sex (Male)	12 (42.9%)	24 (77.4%)	0.008
Age (years)	34 (18–68)	33 (20–78)	0.641
**Recipient**			
Sex (Male)	18 (64.3%)	25 (78.1%)	0.264
Age (years)	57 (35–74)	54 (43–71)	0.870
Indications for LT			0.396
Alcoholic	4	9	
HBV	5	3	
HCV	2	3	
HCC	12	12	
AIH	1	1	
Biliary cirrhosis	0	1	
Budd–Chiari syndrome	0	1	
NBNC	2	0	
PBC	0	1	
PSC	0	1	
Wilson’s disease	2	0	
Retransplantation	0 (0%)	1 (3.1%)	1.000
Type of LT (LDLT)	20 (71.4%)	20 (62.5%)	0.585
MELD	15 (6–40)	14 (6–40)	0.406
Time from LT to study enrollment (mo.)	16.7 (6.9–35.5)	16.6 (6.2–43.5)	0.711
**Efficacy**			
Biopsy-proven acute rejection	0 (0%)	0 (0%)	-
Graft failure	0 (0%)	0 (0%)	-
Death	0 (0%)	0 0%)	-

* LT, liver transplantation; HBV, hepatitis B virus; HCV, hepatitis C virus; HCC, hepatocellular carcinoma; AIH, autoimmune hepatitis; NBNC, non-B non-C; PBC, primary biliary cirrhosis; PSC, primary sclerosing cholangitis; LDLT, living donor liver transplantation; MELD, model for end-stage liver disease.

**Table 2:** Tacrolimus exposure parameters in *CYP3A5* expressors and non-expressors before and after the conversion from twice-daily to once-daily extended-release formulation.

	Before	After	*p*-Value
**Expressor group (*n* = 28)**			
Tacrolimus trough level (Cmin) (ng/mL)	5.0 (2.6–7.9)	3.8 (0.6–5.0)	<0.001
Dose-adjusted Cmin (ng/mL/mg)	1.4 (0.7–3.2)	0.8 (0.4–2.8)	<0.001
Dose/kg-adjusted Cmin (ng/mL/mg/kg)	0.025 (0.008–0.067)	0.015 (0.005–0.048)	<0.001
**Non-expressor group (*n* = 32)**			
Tacrolimus trough level (Cmin) (ng/mL)	4.7 (2.7–7.8)	3.9 (1.8–7.1)	<0.001
Dose-adjusted Cmin (ng/mL/mg)	2.3 (1.0–6.2)	1.7 (0.7–4.7)	<0.001
Dose/kg-adjusted Cmin (ng/mL/mg/kg)	0.038 (0.016–0.115)	0.029 (0.011–0.078)	<0.001

**Table 3:** Pharmacokinetic parameters of tacrolimus stratified according to *CYP3A5* expression.

	Expressor Group (*n* = 5)	Non-Expressor Group (*n* = 5)	*p*-Value
Dose (mg)	4 (3–5)	2 (1–3)	0.016
Weight (kg)	54 (51.3–78.0)	65 (43–82)	0.690
Dose/Weight (mg/kg)	0.077 (0.045–0.097)	0.037 (0.016–0.047)	0.032
AUC in Tac BID (ng·h/mL)	126.3 (98.2–146.9)	107.0 (102.4–126.7)	0.310
AUC in Tac QD (ng·h/mL)	112.5 (81.0–168.6)	96.1 (68.5–131.7)	0.310
Cmin in Tac BID (mg/mL)	3.77 (3.05–4.20)	3.07 (2.76–4.47)	0.421
Cmin in Tac QD (mg/mL)	3.04 (1.85–4.21)	2.59 (1.81–4.47)	0.690
Cmax in Tac BID (mg/mL)	8.20 (4.94–11.54)	9.93 (5.08–10.48)	0.690
Cmax in Tac QD (mg/mL)	9.52 (6.81–11.90)	7.85 (3.54–10.86)	0.151
Dose-adjusted Cmin in Tac BID (ng/mL/mg)	0.918 (0.754–1.050)	1.535 (0920–3.400)	0.056
Dose-adjusted Cmin in Tac QD (ng/mL/mg)	0.680 (0.608–1.053)	1.295 (0.603–2.235)	0.151
Dose-adjusted Cmax in Tac BID (ng/mL/mg)	1.65 (1.58–2.40)	3.79 (3.31–5.16)	0.008
Dose-adjusted Cmax in Tac QD (ng/mL/mg)	2.73 (1.36–3.10)	3.54 (1.85–5.43)	0.310
Dose-adjusted AUC in Tac BID (ng·h/mL/mg)	29.4 (25.3–33.0)	53.5 (35.2–102.4)	0.008
Dose-adjusted AUC in Tac QD (ng·h/mL/mg)	27.0 (21.0–42.2)	48.0 (24.2–68.5)	0.095
Dose/kg-adjusted Cmin Tac BID (ng/mL/mg/kg)	0.015 (0.013–0.018)	0.021 (0.012–0.053)	0.548
Dose/kg-adjusted Cmin Tac QD (ng/mL/mg/kg)	0.013 (0.009–0.014)	0.017 (0.009–0.052)	0.222
Dose/kg-adjusted Cmax Tac BID (ng/mL/mg/kg)	0.032 (0.020–0.046)	0.069 (0.040–0.088)	0.032
Dose/kg-adjusted Cmax Tac QD (ng/mL/mg/kg)	0.038 (0.025–0.052)	0.053 (0.029–0.126)	0.421
Dose/kg-adjusted AUC Tac BID (ng·h/mL/mg/kg)	0.492 (0.423–0.553)	0.716 (0.455–1.600)	0.151
Dose/kg-adjusted AUC Tac QD (ng·h/mL/mg/kg)	0.458 (0.390–0.541)	0.643 (0.379–1.532)	0.421

* AUC, area under the curve; h, hour; Tac, tacrolimus; BID, bis in die (twice daily); QD, quaque die (once daily).

**Table 4:** Adverse events stratified according to *CYP3A5* expression.

	Expressor (*n* = 28)	Non-Expressor (*n* = 32)	*p*-Value
Patients with adverse events	17 (60.7%)	18 (56.3%)	0.796
Adverse events	41	30	
Severity			0.070
Mild	34 (82.9%)	19 (63.3%)	
Moderate	6 (14.6%)	10 (33.3%)	
Severe	1 (2.4%)	1 (3.3%)	
Related to the study drug			0.023
Certain	0 (0%)	0 (0%)	
Probable/likely	5 (12.2%)	0 (0%)	
Possible	5 (12.2%)	0 (0%)	
Unlikely	30 (73.2%)	30 (100%)	
Conditional, unclassified	1 (2.4%)	0 (0%)	
Unassessable/unclassifiable	0 (0%)	0 (0%)	
Subjects with adverse events leading to drug discontinuation	3 (10.7%)	0 (0%)	0.096
